# Supplementary material for: Epistemic Trust, Mistrust and Credulity Questionnaire (ETMCQ) validation in French language: Exploring links to loneliness
Source: PLoS One. 2025 Mar 21;20(3):e0303918. doi: 10.1371/journal.pone.0303918 (PMC11927912; doi:10.1371/journal.pone.0303918)
Supplement: S1 Appendix — (PDF) [file pone.0303918.s001.pdf]

Questionnaire de la confiance, de la méfiance et de la crédulité épistémique.
(Epistemic Trust, Mistrust and Credulity Questionnaire, Campbell 2021)

Pour chacune des phrases suivantes, indiquez dans quelle mesure cela est plus ou moins vrai pour vous, en allant de 1 si vous êtes fortement en désaccord à 7 si vous êtes fortement d'accord, et 4 si vous n'êtes ni d'accord ni en désaccord.

	Question	1	2	3	4	5	6	7
1	Je demande généralement conseil aux gens lorsque j'ai un problème personnel.							
2	Je trouve les informations plus faciles à croire et à assimiler lorsqu'elles viennent de quelqu'un qui me connaît bien.							
3	Je préfère trouver les choses par moi-même sur Internet plutôt que de demander des informations aux gens.							
4	J'ai souvent l'impression que les gens ne comprennent pas ce que je veux et ce dont j'ai besoin.							
5	On me considère souvent comme naïf parce que je crois presque tout ce que les gens me disent.							
6	Lorsque je parle à différentes personnes, je me laisse facilement convaincre par ce qu'elles disent, même si c'est différent de ce que je croyais auparavant.							
7	Parfois, avoir une conversation avec des personnes qui me connaissent depuis longtemps m'aide à avoir de nouvelles perspectives sur moi-même.							
8	Je trouve très utile d'apprendre de ce que les gens racontent de leurs expériences.							
9	Si vous accordez trop de foi à ce que les gens vous disent, vous risquez d'être blessé/e.							
10	Lorsque quelqu'un me dit quelque chose, ma réaction immédiate est de me demander pourquoi il/elle me dit cela.							
11	J'ai trop souvent pris conseil auprès des mauvaises personnes.							
12	On m'a dit que je suis trop facilement influencé/e par les autres.							
13	Si je ne sais pas quoi faire, mon premier réflexe est de demander à quelqu'un dont j'apprécie l'opinion.							
14	Je n'ai pas l'habitude de suivre les conseils que me donnent les autres, même si je pense qu'ils sont probablement judicieux.							
15	Dans le passé, j'ai mal évalué qui croire et j'ai été trompé/e.							
