# Supplementary material for: Epistemic Trust, Mistrust and Credulity Questionnaire (ETMCQ) validation in French language: Exploring links to loneliness
Source: PLoS One. 2025 Mar 21;20(3):e0303918. doi: 10.1371/journal.pone.0303918 (PMC11927912; doi:10.1371/journal.pone.0303918)
Supplement: Attachment — Submitted filename: Response to Reviewers_r.docx [file pone.0303918.s007.docx]

**Response to reviewers**

**Reviewer #1**

I would like to commend the authors for their effort and dedication, which, in my view, have significantly enhanced the quality of the manuscript. The revisions have addressed several key concerns, and the paper is now much improved. I have a few minor comments and suggestions to further refine the manuscript:

1. Lines 74–75: The sentence implies that insecure attachment results in epistemic mistrust. However, as highlighted by Fonagy and colleagues, epistemic credulity could also be an outcome of insecure attachment patterns. Please revise this sentence to reflect both possibilities.

*Answer: Thank you for your comments. Changes were made accordingly.*

2. Line 85: The acronyms for the epistemic stances (ET, EM, and EC) are introduced here. However, in several places later in the manuscript, the full terms are used again (e.g., lines 210–213 and 482). Please ensure consistency in the use of acronyms throughout the manuscript.

*Answer: Changes were made accordingly throughout the manuscript.*

3. Lines 171–173: The phrasing of your third aim is somewhat ambiguous. Please clarify it. Additionally, you mention investigating the mediating role of “epistemic trust,” but I believe you intended to refer to the three epistemic stances.

*Answer: Thank you for your comment, we clarified the sentence.*

4. Lines 286–288: Performing separate CFAs for genders and attachment styles is a precursor to testing configural invariance, but it is not the test itself. Configural invariance involves testing a model with constraints imposed on factor loadings. Furthermore, scalar/weak invariance must be confirmed before conducting between-group mean comparisons. Since you have opted not to test progressive invariance models (which can be justified given the sample size), I recommend removing the section on measurement invariance from the data analysis and results. Instead, please note in the limitations section that the results might be conflated with the potential measurement bias of the ETMCQ due to the absence of invariance testing.

*Answer: Thank you for this proposal, we have removed the section on measurement invariance from the data analysis and results sections, and added the potential bias in the limitation-discussion section (lines 589-591).*

5. Line 340: The manuscript uses the acronym “GFIs” to refer to “Goodness of Fit Indices,” but in some instances, the singular “GFI” is used. This could lead to confusion, as “GFI” in the literature typically refers to a specific fit index. Please standardize the use of this term.

*Answer: We made changes accordingly throughout the manuscript.*

6. Factor Structure, Internal Consistency, and Reliability: The information in this section appears to be presented in the wrong order. Findings regarding model fit indices should be reported first, followed by parameter estimates (e.g., standardized loadings, R-squared values). Rearranging this section would enhance the logical flow and clarity.

*Answer: Thank you for your comment. We rearranged the section accordingly (lines 319-320 and switch between tables 2 and 3).*

**Reviewer #2**

The only shottcoming not picked up is the absence of a reported correlation matrix of all the vriables used in the study which could be incliuded in supplimentary materal. It would help interpretation of findings

*Answer: Thank you for your comment. We have now included a correlation matrix of all the variables used, which is available as a supplementary material (**S4 Appendix: Correlation Matrix).*
