# Supplementary material for: Epistemic Trust, Mistrust and Credulity Questionnaire (ETMCQ) validation in French language: Exploring links to loneliness
Source: PLoS One. 2025 Mar 21;20(3):e0303918. doi: 10.1371/journal.pone.0303918 (PMC11927912; doi:10.1371/journal.pone.0303918)
Supplement: Attachment — Submitted filename: Response to reviewers.docx [file pone.0303918.s006.docx]

**Response to reviewers**

**Journal Requirements:**

When submitting your revision, we need you to address these additional requirements.

1. Please ensure that your manuscript meets PLOS ONE's style requirements, including those for file naming.

*Answer: Corrections were made to meet PLOS ONE’s style requirements to Supplementary Material file’s naming.*

2. We noticed you have some minor occurrence of overlapping text with the following previous publication(s), which needs to be addressed:

https://journals.plos.org/plosone/article?id=10.1371%2Fjournal.pone.0251750

https://pubmed.ncbi.nlm.nih.gov/38156560/?

*Answer: We thank you for your vigilance and made the appropriate corrections accordingly. Thus, the sentence borrowed from Li et al. (2023) (lines 98-102 in the original manuscript) has been modified, and the reference added. Concerning the paragraph derived from Bauer et al. et (2021) (lines 527-537 in the original manuscript), we modified its content and added the reference.*

**Reviewer #1:**

This manuscript provides information on the psychometric properties of the ETMCQ by examining self-report data from a sample of community-dwelling French participants. Statistical analyses focused on CFA, criterion-related validity, reliability analyses, and mediation models.

I have three major concerns about this manuscript:

- There are numerous errors regarding English grammar, sentence structure, and typos. I have specifically mentioned some of them below. Also, I may be wrong, but in some parts, I felt that the text was written in another language and then translated into English. This is a substantial issue hindering the appropriateness of this manuscript. Please have it proofread by a native English speaker. I also believe that the manuscript would significantly benefit from scientific editing.

*Answer: Thank you for your comment, we managed to have the revised manuscript edited and proofread the manuscript by a professional academic editor (Clare Farrar, University College London), hence the significant modifications between the original submission and the revised version.*

- No rationale is provided for the decision-making process regarding improving model fit. After finding an unacceptable fit for the original CFA model, the authors immediately proceeded with item removal. I believe that item removal should be the last resort for improving fit. Strangely, they have not tried to correlate residuals first, and if and when it did not suffice, trying item removal. Additionally, on what basis did the authors remove the lowest-loading item from each subscale (i.e., why not only one item from one subscale? Why not two items from each subscale?)? They mentioned that Campbell et al. (2021) followed the same procedure, but this issue exists for their study too. In short, the statistical and theoretical rationale behind these decisions is unclear.

*Answer: Thank you for your comment. Following some of your further comments, we have revised our process for improving model fit (see Factor structure, internal consistency and reliability section, lines 332-348). We performed our CFA using a polychoric estimation method (Diagonal Weighted Least Squares DWLS), bearing in mind the pros and cons of correlating residuals (in short : risk of unstability versus risk of misspecification) and driven by goodness-of-fit indices (GFI). Also, see the revised Table 3 for adjusted goodness-of-fit measures.*

- The paragraphs about loneliness (in the introduction and the discussion) seem disconnected from the rest of the manuscript, which focuses on ET, attachment, and mentalizing capacity. I wonder if the mediation ideas could be explored more robustly in a separate paper rather than trying to force it to fit in with the broader measurement paper. This decision has led to a rather lengthy and sometimes incoherent manuscript. I think the authors could keep many of these variables in the paper but only utilize them as indicators of the criterion-related validity of the ETMCQ.

*Answer: We appreciate how the sections addressing loneliness in the original manuscript may not have been adequately integrated with the rest of the text. Specifically, the original manuscript may not have fully exposed how loneliness represents a central theoretical pilar to the refined mentalization / epistemic trust framework (Bateman et al. 2023, Fonagy & Allison 2014, Fonagy et al. 2015, Fonagy et al. 2017a, Lorenzini et al. 2018). As a result, the manuscript shifts too quickly and strongly towards parallel research fields on loneliness, particularly the Cacioppo cognitive model, which may have furthered the disconnect. We believe that having a study concomitantly validating the ETMCQ in a new language while exploring its association with loneliness significantly contributes to test the hypotheses upon which the ETMCQ rests,* *and thus enhancing the literature by integrating loneliness as a core dimension of epistemic dysfunctions as attested by other reviewers. In the revised manuscript’s introduction, we thus better articulate how loneliness constitutes crucial theoretical ingredient in the mentalization framework’s latest conceptualization of psychopathology. We moved the elaboration on the Cacioppo cognitive model to the discussion section.*

*Bateman, A., Fonagy, P., Campbell, C., Luyten, P., & Debbané, M. (2023). Cambridge guide to mentalization-based treatment (MBT). Cambridge University Press.*

*Fonagy, P., & Allison, E. (2014). The role of mentalizing and epistemic trust in the therapeutic relationship. Psychotherapy, 51(3), 372.*

*Fonagy, P., Luyten, P., & Allison, E. (2015). Epistemic petrification and the restoration of epistemic trust: A new conceptualization of borderline personality disorder and its psychosocial treatment. Journal of personality disorders, 29(5), 575-609.*

*Fonagy, P., Luyten, P., Allison, E., & Campbell, C. (2017). What we have changed our minds about: Part 1. Borderline personality disorder as a limitation of resilience. Borderline personality disorder and emotion dysregulation, 4, 1-11.*

*Lorenzini, N., Campbell, C., & Fonagy, P. (2018). Mentalization and its role in processing trauma. Approaches to psychic trauma: Theory and practice, 403-422.*

Minor issues:

Introduction: To my understanding, the mechanisms behind the association of ET with relevant constructs (attachment and mentalizing capacity) are not clearly delineated. For instance, how do different epistemic stances develop in early attachment relationships? How does mentalizing open the epistemic highway as an ostensive cue?

*Answer: Thank you for your feedback. In the revised manuscript, we have significantly modified the introduction section to take this point into account. Following other reviewers that considered the introduction section as too broad, we incorporated some elements from the introduction section into the discussion section to better explore the implications of your findings. We hope the clarity and flow of the text are ameliorated.*

Line 33: “investigated” instead of “investigate”.

*Answer: Thank you for pointing out this, corrections were made accordingly.*

Line 37: you should put “the” before “ETMCQ” and “CTQ-SF”

*Answer: Thank you for pointing out this, corrections were made accordingly.*

Line 42: If by “external validity” you mean the association of the ETMCQ with relevant constructs, I believe the appropriate phrase would be “criterion-related validity”. Please correct this throughout the manuscript.

*Answer: Thank you for pointing out this, corrections were made accordingly throughout the manuscript.*

Lines 42-44: “We replicate previous findings that demonstrated epistemic trust’s attachment style related differences, as well as the mediating effect between childhood traumatic experiences and psychopathology.” I believe there are multiple errors in this sentence.

*Answer: Corrected accordingly.*

Line 62: “…a key sources…” Please correct the typo.

*Answer: Corrected accordingly.*

Lines 62-63: “…attributing trust to a key source~~s~~ of social information, that is, to attribute epistemic trust.” I believe “trust” should be replaced with “trustworthiness” here.

*Answer: Thank you for this request for precision. The word “trust” has been replaced by “trustworthiness”.*

Lines 64-66: Please add the page number for your direct quotation.

*Answer: Corrected accordingly.*

Lines 70-73: It may be helpful to expand on the concept of epistemic vigilance in this paragraph, as you have already mentioned it implicitly. Also, this paragraph may be too long and could be divided into two distinct paragraphs.

*Answer: Thank you for your suggestion. We have divided and shortened the paragraph, and added a discussion on epistemic vigilance in the discussion section as proposed by other reviewers.*

Lines 154-156: I wonder about the basis of your hypothesis regarding adequate internal consistency, when it is rather clear from the literature that EM does not show acceptable internal consistency coefficients (Asgarizadeh & Ghanbari, 2024).

*Answer: Corrections were made accordingly (see first paragraph of the discussion section).*

Line 156: “We also anticipate that the ETMCQ scores converge with…” Convergent validity usually refers to the convergence between the scores of the current measure and scores of another measure aimed at the same construct.

*Answer: Corrections were made accordingly.*

Participants: Please describe how you determined the adequate sample size a priori. Regardless, I believe that your sample size is small relative to the current psychometric literature, which should be mentioned as a limitation of this study.

*Answer: Based on Campbell et al. (2021) results indicating a minimal item loading of .406, we have followed Jackson et al. (2013) recommendations supporting that for a CFA with 3 factors and 5 items per factor, a sample size of 200 is sufficient. We have completed the manuscript accordingly. After your comment, we have applied the parameters of the final 12-item model to control for the sample size based on RMSEA as proposed by Kim et al. (2005). Given an RMSEA of .062, a significance of .05 and a power of .80 require a minimal sample size of 170. We believe that the sample size of 302 supports the main results of this study, namely CFA and criterion-related validity. Although, a larger sample is necessary to make comparison by gender and attachment style. We have mentioned this in the limitations’ section of the study.*

*Jackson, D. L., Voth, J., & Frey, M. P. (2013). A note on sample size and solution propriety for confirmatory factor analytic models. Structural Equation Modeling: A Multidisciplinary Journal, 20(1), 86-97.*

*Kim, K. H. (2005). The relation among fit indexes, power, and sample size in structural equation modeling. Structural equation modeling, 12(3), 368-390.*

Lines 178-180: Were these bogus items or instructed-response items (see the reference below)? Also, please explain your screening in more detail (i.e., how many items were included? What was the cutoff for participant exclusion?)

Ward, M. K., & Meade, A. W. (2023). Dealing with careless responding in survey data: Prevention, identification, and recommended best practices. Annual Review of Psychology, 74(1), 577-596. https://doi.org/10.1146/annurev-psych-040422-045007

*Answer : Thank you for this request for information and to provide us with the reference by Ward & Meade (2023). The items bogus items were:*

*- I am currently taking an on-line survey on Prolific*

*- Before brushing my teeth I give a phone call to Victor Hugo*

*- I swim across the Atlantic ocean every morning.*

*We have modified the manuscript accordingly so as to provide more clarity concerning this part of our procedure (Procedure section, first paragraph).*

Additional measures: Please include the internal consistency coefficients of each measure in your study.

*Answer: Internal consistency coefficients for the additional measures CTQ, RFQ, ECR, UCLA, SCL-90 were added in Table 4.*

Data analyses: There is no information regarding the estimation method of the CFA. For instance, did you use ML, MLR, or WLSMV estimation methods? Moreover, you have not mentioned how you intended to examine the parameter estimates of the models (i.e., factor loadings, residual variances [uniquenesses], and R-squared values). These should be included here.

*Answer: We initially used a CFA with Maximum Likelihood estimation assuming continuous variables and with some tolerance for the few items with skewed scores. After your comment on the consistency with the use of Spearman’s correlation, we have used a polychoric correlations estimation method (Diagonal Weighted Least Squares DWLS, Finney & Distefano 2013). Regarding models’ parameter estimates, we have examined standardized item-loadings and their p-value, R^2^, residual variances and covariances, and were driven by item modification indices and models goodness-of-fit indices. We have modified the manuscript accordingly.*

*Finney SJ, Distefano C. Nonnormal and Categorical Data in Structural Equation Modeling. In: Structural Equation Modeling a second course. Information Age Publishing. Charlotte; 2013. p. 439–92*

Lines 235-237: The information regarding the translation procedure is misplaced and should either be presented in the “procedures” subtitle or the “measures/materials” subtitle. Moreover, there are no paragraphs about the ETMCQ in the measures section. Either a concise paragraph should be included, or adequate information about the ETMCQ should be included in the introduction (e.g., its Likert-type scale and the number of items for each subscale).

*Answer: The information regarding the translation procedure has been moved to the end of procedure section. Furthermore, we added a paragraph on the ETMCQ in the measure section.*

Line 243: “…for 243 a, acceptable fit…” Please correct the typo.

*Answer: The typo was corrected.*

Line 243: I wondered why you chose to use “internal coherence” instead of “internal consistency”.

*Answer*: *Corrected accordingly throughout the whole document*.

Line 244: Please also report the Omega coefficients.

*Answer: Omega coefficients were added to the manuscript.*

Lines 245-249: It is currently unclear as to why you have chosen to report both ICC and correlations. If both are reported to examine test-retest reliability, then there is redundancy.

*Answer: Thank you for this demand for clarification. Following Berchtold et al. (2016), our aim was to make the distinction between reliability (i.e. capacity of the scale to produce the same differentiation in participants ranking when they are assessed at 2 different times) and agreement (i.e. capacity to produce the same individuals scores at 2 different times). Reliability is evaluated with test-retest correlation, while agreement requires alternative methods among which intra-class correlation coefficient (ICC) sounds the most accessible theoretically and technically without compromising on methodological requirements (Koo et al. 2016). We amended the manuscript with this clarification.*

*Berchtold, A. (2016). Test–retest : Agreement or reliability? Methodological Innovations, 9, 205979911667287.*

*Koo, T. K., & Li, M. Y. (2016). A Guideline of Selecting and Reporting Intraclass Correlation Coefficients for Reliability Research. Journal of Chiropractic Medicine, 15(2), 155‑163.*

Line 246: “…to control for reliability…” What does it mean to control for reliability? If I am not mistaken, “To control for” is usually used when covariates are involved.

*Answer: Thank you for correcting our misleading use of “to control for”. We re-written the text accordingly.*

Line 249: “Construct validity was established by examining Spearman’s correlations…” Here, you have treated the data as ordinal. As I mentioned earlier, the estimation method for the CFA models is not reported; however, it should match your decision on using the Spearman’s correlation coefficient (i.e., estimation methods that use polychoric correlations and treat data as ordinal, such as the WLSMV).

*Answer*: *Following your comments and computing item responses as ordinal variables, we have revised our CFA using Diagonal Weighted Least Squares estimation method (DWLS), which is based on polychoric correlations and suitable for non-normal distributions and ordinal variables (Finney & Distefano 2013). We have updated the manuscript in methods and results accordingly.*

*Finney SJ, Distefano C. Nonnormal and Categorical Data in Structural Equation Modeling. In: Structural Equation Modeling a second course. Information Age Publishing. Charlotte; 2013. p. 439–92*

Lines 251-252: “…mentalization abilities (RFQ-8), attachment 252 style (ECR-R).” You need to put an “and” in between.

*Answer: Corrections were made accordingly.*

Line 253: Current practice recommendations emphasize the need to establish measurement invariance across the groups prior to conducting multigroup comparisons. In case measurement invariance is not tested, between-group differences (or lack thereof) may be products of methodological artifacts instead of actual differences. This article is an excellent review on the matter:

Putnick, D. L., & Bornstein, M. H. (2016). Measurement invariance conventions and reporting: The state of the art and future directions for psychological research. Developmental Review, 41, 71-90. <https://doi.org/10.1016/j.dr.2016.06.004>

*Answer: Thank you for this helpful comment. Given the complexity of a comprehensive measurement invariance test as proposed by Putnick & Bornstein (2016), we have found reasonable here to check whether the 12-item 4 CR model provides acceptable GFI in each subgroup by gender and by attachment style. GFI of subgroups by gender are given in table below. They indicate that the 12-item model with 4 correlated residuals fits to the observed data in both subgroups of male and female individuals. This supports configural measurement invariance by gender. We have completed the manuscript with a mention of how we have tested measurement invariance and our limits (Factor structure, internal consistency and reliability section, lines 350-354).*

| **Model** | **n** | **CFI** | **TLI** | **SRMR** | **RMSEA** | **Decision** |
| --- | --- | --- | --- | --- | --- | --- |
| 3-F, 12-it, 4 CR | 302 | .978 | .969 | .062 | .062 | Accept |
| Female | 161 | .975 | .965 | .076 | .069 | Accept |
| Male | 141 | .970 | .957 | .077 | .071 | Accept |

Line 254: Please confirm whether invariance is being compared based on gender (i.e., how people self-identify their gender identity) or sex assigned at birth (i.e., biological differences evidenced at birth). If the authors are conflating gender with sex, please change all mentions of gender throughout the manuscript to reflect sex.

*Answer: Thank for this request for precision. Participants were asked to inform of their gender. All descriptions and analyses that mention “Gender” are actually based on gender (Male, Female, no participants gave other answer such as “prefer not to say” or “non-binary”).*

Line 258: Please expand on how you have categorized attachment styles using the ECR-R scores and cite research that previously used the same methodology.

*Answer:* *Regarding attachment styles, we used the categories proposed by Fraley et al. (2000) and categorized individuals replicating the method proposed by Campbell et al. (2021). First we calculated individual’s scores in the 2 attachment dimensions of anxiety and avoidance by averaging their item responses on these 2 dimensions. Based on the whole of individual’s scores, we determined the median anxiety and avoidance scores over our sample. Afterwards, individuals were categorized as with “low” or “high” anxiety, and “low” or “high” avoidance, depending on whether their scores were below or above median scores in anxiety or avoidance. At last, it was possible to replicate Campbell 4 categories of attachment styles : individuals with low anxiety and avoidance were categorized as “Secure”, those with low anxiety and high avoidance as “Dismissing”, with high anxiety and low avoidance as “Preoccupied”, and with high anxiety and high avoidance as “Fearful”. We have reviewed the manuscript accordingly.*

*Fraley, R. C., & Shaver, P. R. (2000). Adult romantic attachment: Theoretical developments, emerging controversies, and unanswered questions. Review of general psychology, 4(2), 132-154.*

Line 266: Please add a proper citation for the Jamovi.

*Answer:* *Reference was added accordingly:* *Jamovi Desktop (Version 2.3.28) and JASP 0.17.1.0* *[Computer Software].*

Results: Correlation results are mainly discussed in terms of whether associations are "significant" or not. Reformulation of this section of the manuscript from the basis of effect size is necessary given the size of these samples and the strong likelihood that the correlations are inflated due to shared method variance. Revising the analytic plan, results, and their interpretation to employ an effect size framework with a clear delineation of benchmarks used to determine meaningful correlations in light of shared method variance would result in a stronger contribution.

*Answer: Thank you for your comment. We have now adopted an effect size framework with clear cut-offs, in line with your suggestions and relevant literature on the topic. Revisions have been made to the data analysis section, results section, and discussion section. It is worthwhile to consult the revised version with corrections to appreciate the modifications made possible by the reviewers’ comments.*

*Podsakoff PM, MacKenzie SB, Lee JY, Podsakoff NP. Common method biases in behavioral research: a critical review of the literature and recommended remedies. Journal of applied psychology. 2003;88(5):879.*

*Sullivan GM, Feinn R. Using effect size—or why the P value is not enough. Journal of graduate medical education. 2012;4(3):279–82.*

*Akoglu H. User’s guide to correlation coefficients. Turkish journal of emergency medicine. 2018;18(3):91–3.*

*Maher JM, Markey JC, Ebert-May D. The other half of the story: effect size analysis in quantitative research. CBE—Life Sciences Education. 2013;12(3):345–51.*

Demographic data: Did you only ask participants regarding their sex/gender and age? If additional information is available (e.g., ethnicity, race, marital status, educational degree, socioeconomic status, sexual orientation, self-reported psychopathology, etc.), please report it here. If not, this is a major limitation.

*Answer: Thank you for your request to better inform readers on the sample characteristics. Participants were asked about their gender (not sex), date of birth, sexual orientation, ethnicity, educational degree, partnership status, and occupational status. We have complemented the manuscript with a table summarizing these information (see Table 1).*

Factor structure, internal consistency and reliability: Please add a table including this information: item descriptives (M, SD, skewness, and kurtosis), standardized factor loadings, residual variances (uniquenesses), and R-squared values for both the 15-item and the 12-item versions.

*Answer: You will now find this data in Table 2 of the revised manuscript.*

Line 278: “…CFI .775; TLI .728…” Equal signs should be added between the index and the value. Please correct this throughout the results.

*Answer: Corrections were made accordingly.*

Line 292: As you mentioned above, correlating residuals requires strong theoretical support (e.g., items measuring the same underlying construct, items having similar wordings, etc.). I believe this sort of support does not exist for correlating residuals of items 13 and 14, as they indicate different subscales.

*Answer: Thank you for this remark. We hope that the answers to preceding comments on model fit improvement and the revised model with 12 items and 4 correlated residuals that does not correlate residuals of items 13 and 14 provide you with receivable explanation. We believe this level of detail is not anymore justified in the manuscript and therefore did not modify the text.*

Lines 292-293: “Internal consistency was acceptable, with Cronbach’s alpha for the entire scale at .701, and for each ETMCQ subscale as follows: Trust .710; Mistrust .690; Credulity .742” In line 245, you proposed a cutoff of .70 for Cronbach’s alpha. Accordingly, Mistrust does not show acceptable internal consistency. The purpose of these cutoffs and benchmarks are to classify results, and adopting a “marginally adequate” stance is unacceptable. Furthermore, the problematic internal consistency of EM is reviewed and discussed in Asgarizadeh & Ghanbari (2024), and your study is not the only one finding poor values. In sum, instead of deeming the internal consistency of EM acceptable, this issue should be mentioned and expanded on in the discussion section.

*Answer: Thank you for stressing this departure from scientific rigor, and for your appreciable comment on the issue on the Epistemic Mistrust subscale consistency. After changes to ordinal variables, we have reviewed and updated internal consistency parameters. Cronbach’s alpha is indeed at .693 for the 12-item model, and at .590 for the EM subscale (still > .70 for Trust and Credulity). This emphasizes the question of the definition of the concept of EM and its assessment. We have modified the text accordingly in the discussion section.*

Line 301: If the EM mean scores are not different across women and men, please report it explicitly rather than how it is currently implied.

*Answer: We now report this explicitly in the revised manuscript line 378.*

Line 304: I guess by “non-parametric” you mean “non-normal”

*Answer: The correction was made accordingly.*

Lines 303-305: I do not understand the relevance of the normality test here. You are reporting it between the mean differences and the correlations. Please clarify your purpose for reporting the normality of distributions, as well as their placement.

*Answer: we made this normality test to select the right method for comparing scores by gender. This phrase was misplaced and has been replaced in the revised manuscript.*

Lines 307-310: Information regarding the model parameters is misplaced and should be reported immediately after model fit indices.

*Answer: We reported it correctly in the revised manuscript.*

Line 314: “* *p* < .05. ** *p* < .01. *** *p* < .001.” There are no asterisks in your figure.

*Answer: Corrections were made accordingly.*

Line 328: “Construct validity” is an umbrella term encompassing several types of validity. Again, I believe the appropriate phrase for association with relevant constructs would be “criterion-related validity”. Please correct this throughout the manuscript.

*Answer: We agree with this comment and have modified the manuscript accordingly.*

Lines 329-330: “Significant correlations between the ETMCQ subscales and related developmental, 330 psychological and psychopathology scales are given in Table 2.” Not all the correlations reported in Table 2 are significant.

*Answer: We modified the sentence.*

Line 331: “…assessed in the CTQ-SF…” It should be replaced with “assessed by the CTQ-SF”.

*Answer: Corrections were made accordingly.*

Line 338: “…certitude subscale… incertitude subscale…” ???

*Answer: These are the RFQ-certainty and RFQ-incertainty subscales. Changes were made accordingly in the manuscript.*

Lines 329-354: I believe these paragraphs have much redundancy. If I am not mistaken, every detail you report here is presented in Table 2. Please shorten this section.

*Answer: We shortened this section accordingly.*

Lines 360-377: The current way of reporting pairwise comparisons is ambiguous and confusing. For instance, you have stated that “Pairwise comparisons showed significant differences between higher score for “Fearful” and “Preoccupied” styles comparing to “Secure” and “Dismissive” ones (ps < .01).” The reader would not understand the significance of each pairwise comparison. This is also not evident in Figure 2. To mitigate this, I suggest adding a table for this section which includes all of the pairwise comparisons for each of the three subscales, as well as reformulating the text.

*Answer: Thank you for your comment.* *We have now presented the pairwise comparisons in a more structured and straightforward way. Given that there are already four tables, we decided not to include an additional one.*

Line 379: “Comparison between to attachment styles…” Please correct the typo.

*Answer: The typo was corrected.*

Line 383: On line 259, you have mentioned “General Linear Model”, and here you have stated “Generalized linear model.” These two have different meanings. Please use the correct term throughout the manuscript.

*Answer: We harmonized our revised manuscript by using the term “generalized linear model” which deals with non-normally distributed data and doesn’t assume a linear relationship.*

Discussion: This section is usually written in the past tense.

*Answer: We changed this section from present to past tense.*

Line 450: intra-class instead of “interclass”

*Answer: Correction was made accordingly.*

Lines 469-474: With a couple of word changes, these sentences are almost fully borrowed from another source, which has not been cited here.

*Answer: Thank you for your vigilance, the omission has been corrected and the source cited (Asgarizadeh et al., 2024), and we rephrased the two sentences.*

Lines 485-486: “…by observing that epistemic trust significantly mediates…” This confuses the reader, as your finding is exactly the opposite.

*Answer: Corrected accordingly.*

Lines 485-502: Previous articles (find below) have discussed that ET subscale may lack validity in non-clinical samples. Your findings mostly support this argument. Please expand on this matter.

Asgarizadeh, A., & Ghanbari, S. (2024). Iranian adaptation of the Epistemic Trust, Mistrust, and Credulity Questionnaire (ETMCQ): Validity, reliability, discriminant ability, and sex invariance. Brain and Behavior, 14(3), e3455. https://doi.org/10.1002/brb3.3455

Asgarizadeh, A., Hunjani, M., & Ghanbari, S. (2023). Do we really need “trust”? the incremental validity of epistemic trust over epistemic mistrust and credulity in predicting mentalizing-related constructs. Asian Journal of Psychiatry, 87, 103688. <https://doi.org/10.1016/j.ajp.2023.103688>

*Answer: We thank you for the identified references, especially the second one, that helped us improve this paragraph by mentioning that several studies using the ETMCQ in non-clinical populations (Asgarizadeh et al. 2023, 2024; Benzi et al. 2023; Liotti et al. 2023; Kampling et al. 2022; Tanzilli et al. 2022) have found little to no significant association between epistemic trust (ET) and related constructs like mentalization, mental health, and personality functioning. We elaborate on this in the discussion section (fourth paragraph) of the revised manuscript.*

Lines 538-557: Another limitation is that you did not examine the convergent validity of the ETMCQ with other measures of ET (see below).

Schröder-Pfeifer, P., Georg, A. K., Talia, A., Volkert, J., Ditzen, B., & Taubner, S. (2022). The Epistemic Trust Assessment—An experimental measure of epistemic trust. Psychoanalytic Psychology, 39, 50-58. https://doi.org/10.1037/pap0000322

Knapen, S., Swildens, W. E., Mensink, W., Hoogendoorn, A., Hutsebaut, J., & Beekman, A. T. F. (2023). The development and psychometric evaluation of the Questionnaire Epistemic Trust (QET): A self-report assessment of epistemic trust. Clinical Psychology and Psychotherapy. <https://doi.org/10.1002/cpp.2930>

*Answer: We added this limitation in the dedicated section.*

**Reviewer #2:**

This manuscript provides a carefully executed replication of the study that developed the ETMCQ, enhancing the literature by integrating loneliness as a dimension of epistemic mistrust. Here are some suggestions to refine the paper further:

1. Introduction: While the introduction is thorough and scholarly, it may be perceived as too broad. Narrowing the focus to literature directly relevant to your hypotheses could streamline the reading experience. Consider relocating some of the currently cited studies to support your discussion of the results.

*Answer: Thank you for your useful comment. We have significantly revised the introduction section and integrated some of its content to the discussion section. The revised manuscript was also edited by a professional academic editor (Clare Farrar, University College London). We believe these adjustments have improved the overall coherence of the manuscript.*

2. Literature Review: It is crucial to comprehensively review prior studies utilizing this instrument, especially those published in this journal. This review should address previous challenges in applying the instrument's structure and any difficulties encountered in replicating its construct validity.

*Answer: Thank you for your feedback, we have made revisions to more thoroughly review in our introduction section Campbell et al. 2021, Liotti et al. 2023, Asgarizadeh et al. 2024. We further review the Argentine validation by Rodriguez Quiroga et al. 2024 and the German validation by Weiland et al. 2024 published in October and November 2024 respectively in PLOS One. Both findings and challenges were addressed.*

3. Clarity of Hypotheses: The hypotheses should be stated more clearly. Additionally, consider using shorter paragraphs for ease of reading, though this may be a matter of personal preference.

*Answer: Thank you for your feedback, we have made revisions to enhance the clarity of the hypotheses section accordingly at the end of the introduction section (Aims and Hypothesis section).*

4. Statistical Approach: The statistical methods used are suitable; however, the application of the ECR-R as a categorical measure rather than a continuous one raises questions. The section on construct validity is compelling and supports the validity of this French version of the instrument. The mediation models align with prior studies, and the novel link between loneliness, psychopathology, and mistrust is intriguing.

*Answer: Thank you for your comments. Regarding the application of the ECR-R as a categorical measure, as already mentioned in a previous response to a reviewer we used the categories proposed by Fraley et al. (2000) and categorized individuals replicating the method proposed by Campbell et al. (2021).*

*Fraley, R. C., & Shaver, P. R. (2000). Adult romantic attachment: Theoretical developments, emerging controversies, and unanswered questions. Review of general psychology, 4(2), 132-154.*

5. Discussion: The discussion reiterates findings extensively but falls short conceptually, especially compared to the comprehensive introduction. There seems to be minimal effort to interpret the findings within a broader psychological model. I recommend incorporating some elements from the introduction into the discussion to better explore the implications of your findings. Furthermore, the limitations section appears perfunctory. It would benefit from a more thorough exploration of the inherent constraints of self-report questionnaires, such as the potential for correlations driven by similar reporting styles rather than a genuine association between constructs.

*Answer: Thank you for your comment which guided some of the re-writing of the discussion section. To address this specific comment, we have revised the discussion to better integrate elements from the introduction, allowing us to explore the implications of our findings in greater depth. We believe this enhances the conceptual richness of the discussion and provides a more comprehensive understanding of how our results fit within existing psychological models and hypothesis. We also reduced some content in the discussion section to avoid excessive repetition of our findings.*

*Regarding the limitations section, we acknowledge your concern about its initial brevity. We have expanded this section to provide a more thorough examination of the limitations. We believe these revisions add critical nuance to our interpretation of the data and present a more balanced view of our study’s strengths and limitations.*
